# Field evaluation of malaria malachite green loop-mediated isothermal amplification in health posts in Roraima state, Brazil

**DOI:** 10.1186/s12936-019-2722-1

**Published:** 2019-03-25

**Authors:** Heather M. Kudyba, Jaime Louzada, Dragan Ljolje, Karl A. Kudyba, Vasant Muralidharan, Joseli Oliveira-Ferreira, Naomi W. Lucchi

**Affiliations:** 1 Center for Tropical and Emerging Global Diseases (CTEGD), University of Georgia, Athens, USA; 2 Federal University of Roraima, Boa Vista, Roraima Brazil; 3 Malaria Branch, Division of Parasitic Diseases and Malaria, Centers for Disease Control and Prevention, Atlanta, USA; 4 Institute Oswaldo Cruz-Fiocruz, Rio de Janeiro, Brazil

**Keywords:** Malaria, *Plasmodium*, Malachite green loop-mediated isothermal amplification, Diagnosis

## Abstract

**Background:**

Microscopic detection of malaria parasites is the standard method for clinical diagnosis of malaria in Brazil. However, malaria epidemiological surveillance studies specifically aimed at the detection of low-density infection and asymptomatic cases will require more sensitive and field-usable tools. The diagnostic accuracy of the colorimetric malachite green, loop-mediated, isothermal amplification (MG-LAMP) assay was evaluated in remote health posts in Roraima state, Brazil.

**Methods:**

Study participants were prospectively enrolled from health posts (healthcare-seeking patients) and from nearby villages (healthy participants) in three different study sites. The MG-LAMP assay and microscopy were performed in the health posts. Two independent readers scored the MG-LAMP tests as positive (blue/green) or negative (clear). Sensitivity and specificity of local microscopy and MG-LAMP were calculated using results of PET-PCR as a reference.

**Results:**

A total of 91 participants were enrolled. There was 100% agreement between the two MG-LAMP readers (Kappa = 1). The overall sensitivity and specificity of MG-LAMP were 90.0% (95% confidence interval (CI) 76.34–97.21%) and 94% (95% CI 83.76–98.77%), respectively. The sensitivity and specificity of local microscopy were 83% (95% CI 67.22–92.66%) and 100% (95% CI 93.02–100.00%), respectively. PET-PCR detected six mixed infections (infection with both *Plasmodium falciparum* and *Plasmodium vivax*); two of these were also detected by MG-LAMP and one by microscopy. Microscopy did not detect any *Plasmodium* infection in the 26 healthy participants; MG-LAMP detected *Plasmodium* in five of these and PET-PCR assay detected infection in three. Overall, performing the MG-LAMP in this setting did not present any particular challenges.

**Conclusion:**

MG-LAMP is a sensitive and specific assay that may be useful for the detection of malaria parasites in remote healthcare settings. These findings suggest that it is possible to implement simple molecular tests in facilities with limited resources.

## Background

Malaria is a devastating disease that remains a major global health burden. The illness arises from infection with parasites of the genus *Plasmodium*. Cases of the most significant morbidity and mortality in humans are caused by the most prevalent species, *Plasmodium vivax* and *Plasmodium falciparum*. *Plasmodium ovale* and *Plasmodium malariae* also cause human malaria, but the infections are typically associated with milder symptoms. In 2017, an estimated 219 million cases of malaria occurred worldwide [1]. Most malaria cases in 2017 were reported in sub-Saharan Africa (200 million, 92%). The WHO Region of the Americas recorded a rise, largely due to increases in malaria transmission in Brazil, Nicaragua, and Venezuela [1]. In Brazil, the vast majority of malaria cases are concentrated in the Brazilian Amazon Region. The State of Roraima in Brazil is located in the Amazon region in the far north on the border with Venezuela and Guyana. In 2017, Roraima reported 11,966 cases of malaria, which was a 44% increase compared to 2016 (8307) [2]. Based on data from the Brazilian Secretariat of Health Surveillance, 50% of patients seen in the State of Roraima were residents of Venezuela and Guyana. There is frequent movement of the population and vectors in the border region, and access to preventive healthcare in Venezuela and Guyana is limited. Control of malaria in Roraima is endangered and the border area is vulnerable to malaria outbreaks and epidemics.

One of the challenges for malaria surveillance and control programmes is the timely identification of low-density infections not detected by the routine diagnostic tests: microscopy and standard rapid diagnostic tests (RDTs). Currently, the primary method used in Brazil for the diagnosis of malaria is microscopy of a Giemsa-stained thick or thin blood smear, but there are limitations of microscopy, including inability to detect very low density (sub-microscopic) parasitaemia, occasional misdiagnosis of mixed-species infection, and the fact that it is time consuming [3–7]. A majority of sub-microscopic infections are asymptomatic. Individuals who are asymptomatic do not seek treatment resulting in a population of individuals with persistent infections, capable of transmitting malaria in the population, (reviewed in [8]). It is important to identify and treat persons with these low-level parasitaemia during malaria epidemiological surveys. Furthermore, the elimination of malaria will require active case detection in low transmission areas as well as the ability to detect sub-microscopic infections [9]. There is a need to develop and validate sensitive diagnostic tools. Molecular-based diagnostic tools provide more sensitive and specific methods for detecting *Plasmodium* infections than microscopy and RDTs. For a ‘significant improvement’ over expert microscopy, it is recommended that molecular tests be at least 1 log more sensitive than microscopy; preferably have a detection limit of 2 parasites/μL or fewer [10]. The use of molecular-based diagnostic tools in research and in epidemiological surveys has expanded in recent years. However, their use is limited to laboratories with more sophisticated facilities, due to the requirement for specialized equipment and technical expertise. Simpler molecular tests, such as the loop-mediated isothermal amplification (LAMP) assays, promise to facilitate the use of molecular tests even in facilities with limited resources [11–15].

As recently reviewed [16], several malaria LAMP-based assays have been described to date. Many of these have excellent diagnostic performance, e.g., detecting as few as 1 parasite/μL (*illumi*gene LAMP), or 1–5 parasites/μL (EIKEN LAMP), however, they are not without limitations, which include the requirement for additional equipment for read-out, the limited number of samples tested per run, and the fact that some are capable of detecting malaria parasites at genus level only. Recently, the development of a malaria malachite green loop-mediated isothermal amplification (MG-LAMP) as a LAMP method for diagnosing *Plasmodium* infection was reported [17]. Three factors make the MG-LAMP assay appealing: (1) performance of the MG-LAMP assay requires only a small portable heat block and mini-centrifuge; (2) it is a colorimetric assay that does not require any special read-out equipment; and, (3) the heat block used has a 38-sample capacity allowing for the testing of many samples at once, with the potential for use in large-scale studies. To date, only two other high through-put (HTP) colorimetric malaria LAMP assays have been described [18, 19].

In this study, the performance of the MG-LAMP assay was tested in health posts of three municipalities of Roraima, Brazil using freshly isolated patient samples. The MG-LAMP diagnosis was compared to results provided by local microscopists at the sites of study. The sensitivity and specificity of MG-LAMP performed in these remote health posts, with limited laboratory infrastructure, were compared to that of a real-time PCR (PET-PCR) [20] assay.

## Methods

### Collection of clinical samples

This prospective study was carried out between July and August 2017 in malaria heath posts in three municipalities of Roraima, Brazil (Boa Vista, Pacaraima, Rorainopolis). All patients attending the health posts for malaria screening and treatment were eligible to be enrolled in the study. In addition, healthy controls were enrolled from houses near the health posts. Blood samples were obtained from all enrolled patients by venipuncture. Enrolled patients were tested for malaria by a trained local microscopist using 10% Giemsa-stained thick blood smear, and the diagnosis and parasitaemia level were recorded for each patient. Additionally, all consenting patients filled out a clinical questionnaire that addressed whether the patient had symptoms, their age, gender, residence, and whether they had prior *Plasmodium* infections.

### LAMP logistics

Blood sample collection and processing, microscopy, DNA extraction, and MG-LAMP assays were all performed in the malaria health posts in Roraima by a USA-based graduate student with training in molecular biology but with no field experience. Two laboratory technicians with no previous experience with LAMP were trained to read the MG-LAMP results. To simplify the MG-LAMP procedure, a three-component ready-to-use kit was used: component I contained all the necessary reaction components for the assay (LAMP buffer: 40 mM Tris–HCL pH 8.8, 20 mM KCl, 16 mM MgSO_4_, 20 mM (NH_4_)SO_4_, 0.2% Tween-20, 0.8 M Betaine, and 2.8 mM of dNTPs and the primers (stored in a 4 °C refrigerator); component II contained the Bst polymerase (stored at − 20 °C), and component III contained 0.2% malachite green dye. To perform the assay, 13.8 µL of Component I was mixed with 0.8 µL of the Bst polymerase and 0.4 μL of the malachite green dye for a final concentration of malachite green of 0.008%. Five µL of DNA template was added and the tubes were placed in the preheated heat block.

### DNA extraction

The DNA extraction was performed in small rooms within the health posts. DNA was extracted from 200 μL of whole blood using the QIAamp DNA Mini Kit (Qiagen Inc, Chatsworth, CA, USA). The manufacturer’s provided DNA extraction protocol was slightly modified in that all of the spins were performed at 2000 g using a mini-centrifuge (Myfuge™) that was easily transported in the field setting.

### LAMP method

All samples were screened for *Plasmodium* using the genus assay as described previously [17] in a final reaction volume of 20 μL. Samples were incubated for 1 h at 63 °C in a mini heat block (GeneMate, Bioexpress, Utah, USA) to amplify the DNA. Following the 1-h incubation, samples were removed from the heat block and allowed to cool for 15 min, the results were then scored by two independent readers as being positive (light blue/green) or negative (clear/colourless). Positive and negative controls were included during each run using *P. falciparum* 3D7 DNA or nuclease-free water, respectively. *Plasmodium falciparum* and *P. vivax* species-specific MG-LAMP assays were carried out on all samples that were positive by the genus assay. These assays were performed using the 3-component ready-to-use in-house kits prepared using previously published *P. falciparum* and *P. vivax* primers [21, 22]. Each reaction contained 5 μL of isolated DNA in a final reaction volume of 20 μL. Positive controls included a *P. falciparum*-positive sample and a *P. vivax*-positive sample. Nuclease-free water was included as a negative control.

### PET-PCR method

DNA samples were shipped to the malaria branch laboratory at the CDC using cold packs. *Plasmodium* genus-specific PET-PCR was performed in duplicate as described previously except that 5 μL of DNA was used instead of 2 μL [20]. The reactions contained 2× TaqMan Environmental Master Mix 2.0 (Applied Biosystems, Foster City, CA, USA), 250 nM of Genus forward Primer and FAM-Genus reverse primer, and 5 μL of isolated DNA for a final volume of 20 μL. The PET-PCR reaction was run using an Agilent Mx3005pro thermocycler (Agilent Technologies, Santa Clara, CA, USA) using the following cycling parameters: 15 min initial hot-start at 95 °C followed by 45 cycles of denaturing at 95 °C for 20 s, annealing at 63 °C for 40 s, and an extension of 30 s at 72 °C. A positive and negative control, 3D7 and nuclease-free water, respectively, were included in each run. Samples were designated as positive if they had a threshold cycle (Ct) value below 40.0 and negative if they had no Ct value or Ct values above 40.0. Species-specific PET-PCR was performed in duplicate on all samples that were positive by the genus specific PET-PCR, using species-specific primers. *P. falciparum* and *P. ovale* PET-PCR primers have been used and verified previously [20, 23]. *P. malariae* and* P. vivax* PET-PCR primers can be found in Table 1. Two duplex reactions were set up to detect *P. ovale* together with *P. falciparum* and *P. malariae* together with *P. vivax.* The duplexed reactions were 20 μL containing 2× TaqMan Environmental Master Mix 2.0 (Applied Biosystems), 250 nM of FAM-*P. ovale* forward primer, 250 nM *P. ovale* reverse primer, 250 nM of *P. falciparum* forward primer, 125 nM of HEX-*P. falciparum* reverse primer, 250 nM *P. malariae* forward primer, 250 nM FAM-*P. malariae*, 125 nM *P. vivax* forward primer, 125 nM HEX-*P. vivax* reverse primer and 5 μL of isolated DNA. Reactions were run using the same cycling conditions as the Genus PET-PCR. Positive controls consisting of samples with known *Plasmodium* species and nuclease-free water as a negative control were included in each run.

**Table 1:** PET-PCR primers utilized in the evaluation

Primer	Sequence
*P. vivax* Forward	5′-ACT GAC ACT GAT GAT TTA GAA CCC ATT T-3′
HEX-*P. vivax* Reverse	5′-agg cgc ata gcg cct ggT GGA GAG ATC TTT CCA TCC TAA ACC T-3′
*P. malariae* Forward	5′-AAGGCAGTAACACCAGCAGTA-3′
FAM-*P. malariae* Reverse	5′-agg cgc ata gcg cct ggTCCCATGAAGTTATATTCCCGCTC-3′

HEX-labelled: based on the *plasmepsin* gene; FAM-labelled: based on *dihydrofolate reductase*-*thymidylate synthase* (DHFR-TS) gene

### Statistical analyses

The percentage specificity and sensitivity were calculated as follows: Sensitivity  =  true positives/(true positives + false negatives) × 100. Specificity  =  true negatives/(true negatives + false positives) × 100. In addition, 95% Confidence Intervals (95% CI) for both sensitivity and specificity were calculated. The agreement between the human readers and diagnostic tests was assessed by calculating the Kappa coefficients. 95% CIs were calculated using MEDCALC^®^ and GraphPad.

## Results

### Patient enrolment

**Fig. 1:**
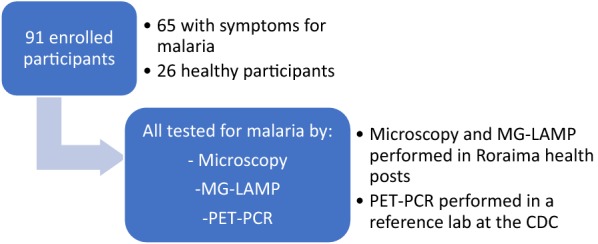
Summary of enrolled patients and sample processing

A total of 91 participants were enrolled during the 2 months of the study: 65 patients presenting with malaria symptoms (axillary temperature ≥ 37.5 °C) at the health posts and 26 healthy participants from nearby villages (Fig. 1). None of the 26 healthy participants exhibited any symptoms of malaria. Of the 91 enrolled participants, 86 (94.5%) reported having had previous malaria infections while 4 (4.4%) had no previous malaria, and 1 (1.1%) did not provide this information.

### Agreement between human readers for the MG-LAMP assay

Overall, performing the MG-LAMP in this setting did not present any particular challenges. Two independent human readers scored the MG-LAMP tests as positive or negative. There was 100% agreement between the two readers (Kappa = 1).

### Overall results of microscopy, MG-LAMP, and PET-PCR

**Fig. 2:**
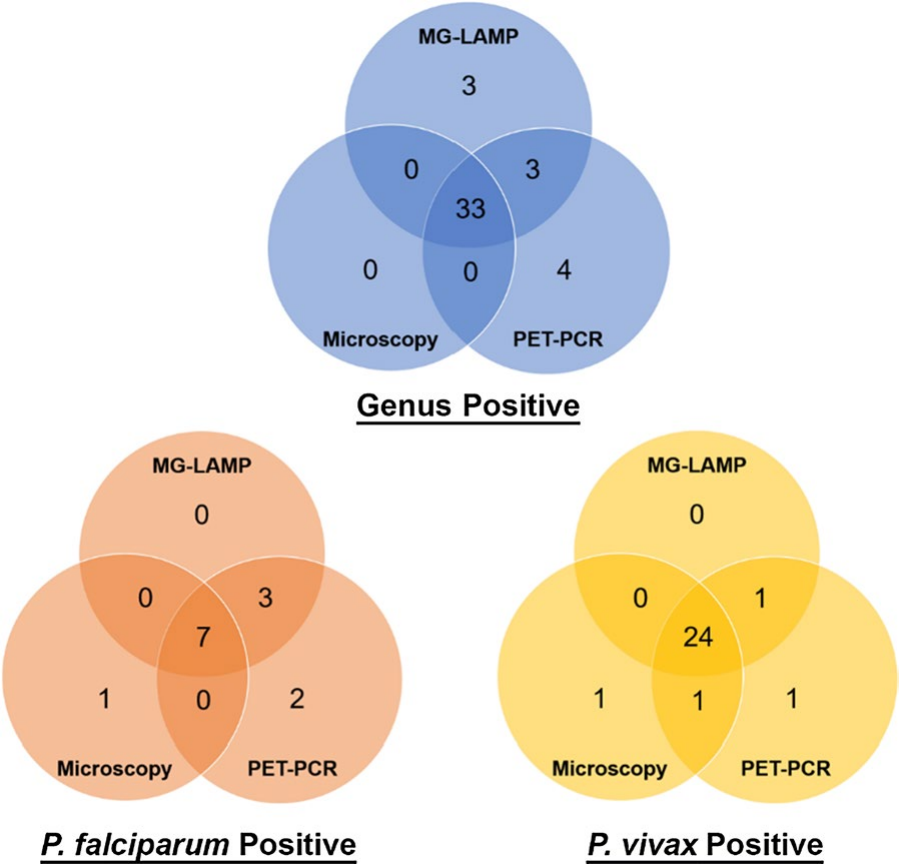
Summary of positive results by microscopy, MG-LAMP and PET-PCR

Of the 91 samples, 33 (36%) were malaria positive by microscopy, 39 (43%) were positive by MG-LAMP, and 40 (44%) were positive by PET-PCR (Fig. 2). All samples were negative for *P. malariae* and *P. ovale*.

### Specificity and sensitivity of MG-LAMP and microscopy compared to PET-PCR

The sensitivity and specificity of the MG-LAMP assays and microscopy were calculated using PET-PCR as a reference test (Table 2).

**Table 2:** Sensitivity and specificity of MG-LAMP and microscopy using PET-PCR as a reference

	Method	Sensitivity	Specificity
Genus^a^	Microscopy (n = 91)	83% (95% CI 67.22–92.66%)	100% (95% CI 93.02–100.00%)
	MG-LAMP (n = 91)	90% (95% CI 76.34–97.21%)	94% (95% CI 83.76–98.77%)
*P. falciparum*	Microscopy (n = 91)	64% (95% CI 93.02–100.00%)	99% (95% CI 93.23–99.97%)
	MG-LAMP (n = 39)	82% (95% CI 48.22–97.72%)	100% (95% CI 95.49–100.00%)
*P. vivax*	Microscopy (n = 91)	83% (95% CI 65.28–94.36%)	98% (95% CI 91.20–99.96%)
	MG-LAMP (n = 39)	90% (95% CI 73.47–97.89%)	100% (95% CI 94.13–100.00%)

^a^Samples which were negative for genus were considered to be negative for species in the sensitivity and specificity calculations

### Agreement of MG-LAMP with PET-PCR

The data show that *Plasmodium* genus assay for MG-LAMP and PET-PCR agreed 92.3% of the time (Kappa = 0.84, 95% CI 0.732–0.955). When comparing *P. falciparum* and *P. vivax* MG-LAMP and PET-PCR assays, there was 97.8% (Kappa = 0.89, 95% CI 0.735–1.000) and 96.7% (Kappa = 0.92, 95% CI 0.839–1.000) agreement between the two tests, respectively.

### Detection of mixed infections

Microscopy detected one mixed *P. falciparum* and *P. vivax* infection, which was detected to be a *P. falciparum* only infection by both the MG-LAMP and PET-PCR assays. There were six mixed infections detected by PET-PCR; two of these were also identified by MG-LAMP but none was identified by microscopy. In the four cases where the MG-LAMP did not detect the mixed infections identified by the PET-PCR, the Ct values were high, suggesting low parasite density infections (Table 3).

**Table 3:** Detection of mixed infections by PET-PCR, MG-LAMP and microscopy

Sample	Microscopy diagnosis	MG-LAMP diagnosis	PET-PCR diagnosis	PET-PCR CT value for *P. falciparum*	PET-PCR CT value for *P. vivax*
PC121	*P. vivax*	Mixed	Mixed	22.68	28.50
PC123	*P. falciparum*	Mixed	Mixed	26.56	39.90
BV237	*P. vivax*	*P. vivax*	Mixed	35.10	29.12
BV217	*P. vivax*	*P. vivax*	Mixed	36.24	32.23
BV239	*P. vivax*	*P. falciparum*	Mixed	31.37	35.38
BV241	*P. falciparum*	*P. falciparum*	Mixed	29.43	39.92
BV240	Mixed^a^	*P. falciparum*	*P. falciparum*	37.82	No Ct

^a^Only one *P. vivax* parasite was seen by microscopy for this sample

### Detection of parasitaemia in asymptomatic patients

Of the 26 enrolled healthy participants, five were positive for *Plasmodium* by MG-LAMP and three were positive for *Plasmodium* by PET-PCR assay. None of these was positive by microscopy (Table 4). Four of the five cases that were positive by MG-LAMP were positive only at genus level and the infecting species could not be determined (Table 4). Two of these samples were positive by both MG-LAMP and PET-PCR, one only at genus level.

**Table 4:** Results of MG-LAMP and PET-PCR in asymptomatic patients

Sample	Microscopy diagnosis	MG-LAMP diagnosis	PET-PCR genus (Ct value)	PET-PCR *P. vivax* (Ct value)	PET-PCR *P. falciparum* (Ct value)
RR09	Negative	Genus only	Negative (40.70)	Negative (No Ct)	Negative (No Ct)
RR10	Negative	Genus only	Negative (41.76)	Negative (No Ct)	Negative (No Ct)
RR37^a^	Negative	*P. vivax*	Positive (32.74)	Positive (35.96)	Negative (No Ct)
RR41^a^	Negative	Genus only	Positive (38.76)	Negative (41.99)	Negative (No Ct)
RR42	Negative	Genus only	Negative (40.74)	Negative (41.69)	Negative (No Ct)
RR53	Negative	Negative	Positive (34.99)	Positive (39.09)	Negative (No Ct)

^a^Samples shown to be positive by both MG-LAMP and PET-PCR

### Discordant results

Seven samples were found to have discordant results among the three tests (Table 5). Four of these samples were negative by microscopy and MG-LAMP but positive by PET-PCR. Three of these samples were positive by PET-PCR genus test and negative by species tests, while one was positive by PET-PCR *P. vivax* (Table 5). In these four cases, the Ct values by PET-PCR were all above 35.0. Three samples yielded a positive MG-LAMP genus test but were negative for the MG-LAMP *P. falciparum* and *P. vivax* tests and by both microscopy and PET-PCR (Table 5).

**Table 5:** Summary of discordant results

Sample	Microscopy diagnosis	MG-LAMP genus diagnosis	PET-PCR genus (Ct value)	PET-PCR *P. vivax* (Ct value)	PET-PCR *P. falciparum* (Ct value)
RR53	Negative	Negative	Positive (34.99)	Positive (39.09)	Negative (No Ct)
BV235	Negative	Negative	Positive (35.78)	Negative (No Ct)	Negative (No Ct)
RR01	Negative	Negative	Positive (37.96)	Negative (No Ct)	Negative (No Ct)
BV236	Negative	Negative	Positive (39.34)	Negative (No Ct)	Negative (No Ct)
RR09	Negative	Positive	Negative (40.70)	Negative (No Ct)	Negative (No Ct)
RR10	Negative	Positive	Negative (41.76)	Negative (No Ct)	Negative (No Ct)
RR42	Negative	Positive	Negative (40.74)	Negative (41.69)	Negative (No Ct)

## Discussion

The findings presented in this study demonstrate the accuracy of the MG-LAMP as a malaria diagnostic test in remote health posts in a malaria-endemic country. Importantly, these data demonstrate that the MG-LAMP is sensitive at identifying infections not detectable by microscopy. Additionally, the results establish that this assay, like the PET-PCR assay used as a reference test in this study, is capable of detecting mixed infections that microscopy missed. However, the MG-LAMP assay missed four positive samples and four mixed infections detected by PET-PCR. These missed infections were all shown to be of much lower parasite densities (based on the high Ct values (between 35 and 39) in the PET-PCR assay. Extrapolation using previously obtained PET-PCR data shows that a Ct value of 35.0 corresponds to about 16 parasites/μL [20], therefore, the missed samples likely had parasite densities of about 16 parasites/μL (3 samples) and below (5 samples). While a detection limit of 16 parasites/μL is much better than that for routine microscopy, it is below the detection limits of many PCR-based assays and some previously published LAMP-based assays, which claim detection limits below 16 parasites/μL. Previously, the malaria MG-LAMP assay was shown to have a limit of detection of 1–8 parasites/μL [17] using quantified standard curves, however, this limit of detection did not hold when the assay was performed in a field setting. More sensitive MG-LAMP primers or a change in assay conditions may be required to achieve the same level of diagnostic accuracy as the PET-PCR assay in field settings. In addition, there were three cases where MG-LAMP yielded a positive genus result, while microscopy and PET-PCR were negative. It is likely that these are false positives by the MG-LAMP assay, however, one cannot rule out that these are indeed true positives missed by PET-PCR, a phenomenon that has been observed before in evaluation studies using low-density infection samples [11, 12].

While PCR-based assays, such as PET-PCR, have superior sensitivity for diagnosing low-density infections, they are far more complicated procedurally compared to the MG-LAMP, as they require costly equipment and supplies. The MG-LAMP assay evaluated in this study can be performed using a small portable heat block and mini-centrifuge and does not require any special read-out equipment since it is a colorimetric assay. It is an appealing test for use in resource-limited facilities. In addition, it has a 38-sample capacity allowing for HTP testing and therefore has the potential for use in large-scale studies. Further investment in refining simple molecular tests to increase sensitivity would allow them to be used in resource-limited settings for the detection of low-density infections.

A limitation of the current format of the MG-LAMP is the fact that the LAMP buffers and polymerase require cold chain, which is not ideal in more resource-limited settings. Currently, there are two available malaria LAMP assays that do not require a cold chain: the EIKEN LAMP and *illumi*gene LAMP, but each of these have limitations, reviewed in [16]. For example, the *illumi*gene LAMP assay is a genus-specific test only and is only capable of testing 10 samples per run. Elimination of the need for a cold chain will be required if the MG-LAMP assay is to be used in settings without a laboratory. However, in facilities similar to the health posts used in this study, the current format of MG-LAMP assay can be performed. The use of DNA extracted using commercially available blood kits should be avoided as this adds extra steps and cost to the test; the use of boil-and-spin DNA isolation should be further explored in future field studies.

Although healthy participants were enrolled in an effort to estimate the ability of the MG-LAMP to detect asymptomatic parasitaemia, the number of healthy participants was too low to draw firm conclusions.

## Conclusion

Overall, MG-LAMP evaluated in this study provided a portable, sensitive and specific assay for the detection of malaria parasites in a remote health clinic in Brazil when compared to microscopy. However, the current format of the assay was not sensitive enough to be recommended for detection of low-density infections and improvements will be required to enhance its sensitivity and if possible, to make it a more field usable tool that does not require a cold-chain.

## Abbreviations

MG-LAMP: malachite green loop-mediated isothermal amplification
RDT: rapid diagnostic test
HTP: high through-put
PET-PCR: photo-induced electron transfer-polymerase chain reaction
CI: confidence interval
CT: cycle threshold
